# Primary Neuroendocrine Tumor of the Breast: A Case Report and Review of Current Guidelines

**DOI:** 10.7759/cureus.73151

**Published:** 2024-11-06

**Authors:** Abigail Cuevas, Fauzia Khattak

**Affiliations:** 1 College of Medicine, University of Illinois at Chicago, Rockford, USA; 2 Medical Oncology, UW (University of Wisconsin) Health, Rockford, USA

**Keywords:** breast, case report, imaging, immunohistochemistry staining, metastases, neuroendocrine tumor, primary neoplasm, treatment resistance

## Abstract

Primary neuroendocrine tumor of the breast is a rare subtype of breast carcinoma that most commonly affects postmenopausal women in their sixth or seventh decade of life. We report a case of a 52-year-old female who presented to our clinic with concerns about a self-palpable mass involving her left breast that had not been detected on a routine mammogram five months prior. Upon clinical exam and diagnostic workup comprising imaging, a tissue biopsy, and immunostaining, she was found to have primary high-grade neuroendocrine carcinoma of the breast with necrosis. Subsequently, management of her case involved a first-line systemic chemotherapy regimen, including carboplatin with etoposide and atezolizumab. Less than two months after beginning therapy, she had undergone three cycles but did not show any significant clinical response, requiring further workup. This case demonstrates the challenges faced with treating such a rare subtype of breast carcinoma and the necessity of documenting other similar cases for improved understanding and patient outcomes.

## Introduction

Primary neuroendocrine tumors (NETs) of the breast are a rare subtype of breast carcinomas that predominantly affect postmenopausal women in the sixth to seventh decade of life. Patients commonly have stage 2 disease at presentation and a higher likelihood of regional lymph node metastases in comparison to patients with invasive ductal carcinoma, not otherwise specified (IDC-NOS) [1]. These tumors are further classified into well-differentiated NETs, poorly differentiated NETs, and invasive breast carcinoma with neuroendocrine features, based on the expression of neuroendocrine markers synaptophysin and chromogranin [2]. Importantly, the principal differential diagnosis for primary NETs is metastatic NET from a site outside of the breast, making it imperative that a CT of the chest and abdomen or a PET scan be performed prior to treatment [2,3].

Clinically, NETs of the breast are difficult to describe, as specific neuroendocrine markers are not regularly checked with breast cancer diagnosis. The prognosis of NETs of the breast remains controversial as they were previously thought to have a better prognosis than that of IDC-NOS but have more recently been suggested to have worse long-term outcomes than non-endocrine breast carcinoma of the same stage [3].

We report a case of a 52-year-old woman with primary high-grade neuroendocrine carcinoma of the left breast. Following three rounds of first-line systemic chemotherapy, she showed no clinical improvement and subsequently underwent left lumpectomy with plans for post-surgical chemotherapy and radiation. Due to her rapid decline, she underwent repeat imaging, which showed evidence of metastases throughout her left breast, liver, and brain. This case demonstrates the aggressive nature of primary neuroendocrine carcinoma of the breast and the rapid decline that may follow an otherwise appropriate treatment regimen. Importantly, this illustrates the need for further development of NET-specific guidelines so that patients like ours may have a more favorable prognosis upon presentation.

## Case presentation

**Figure 1:**
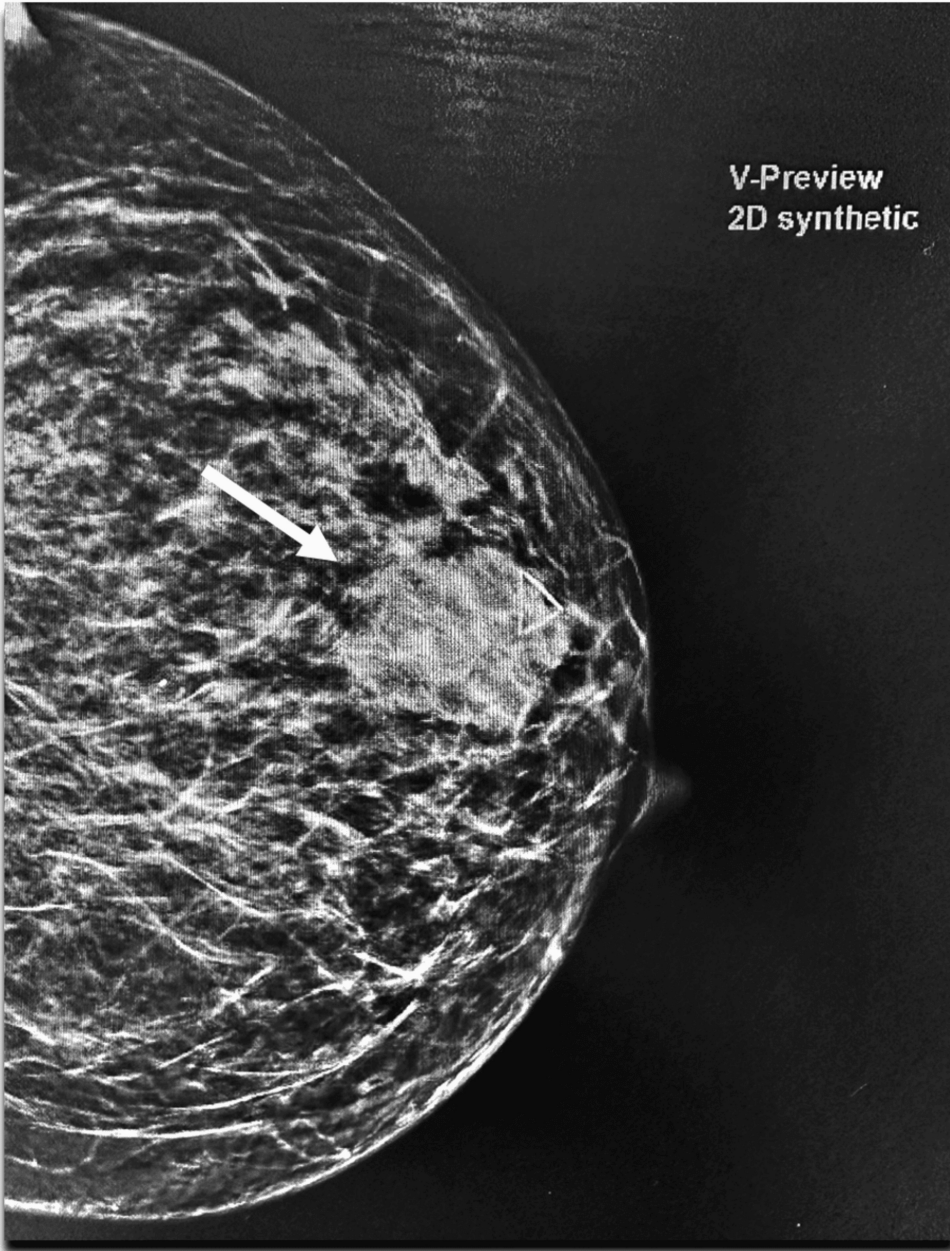
Diagnostic 3D mammogram: mass at the 12:30 position, 4 cm from the nipple measuring 4.1 x 2.5 x 3.4 cm.

**Figure 2:**
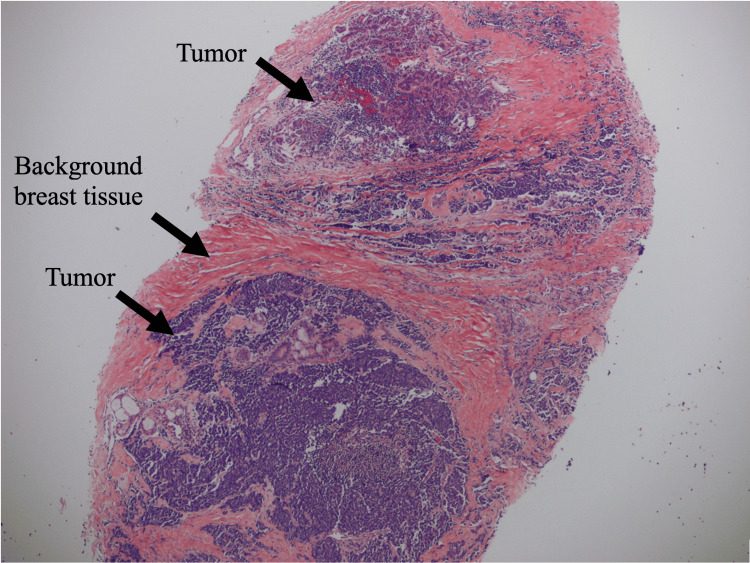
Histopathologic exam of biopsy specimen taken at low power (4x), highlighting breast tissue with tumor infiltration.

**Figure 3:**
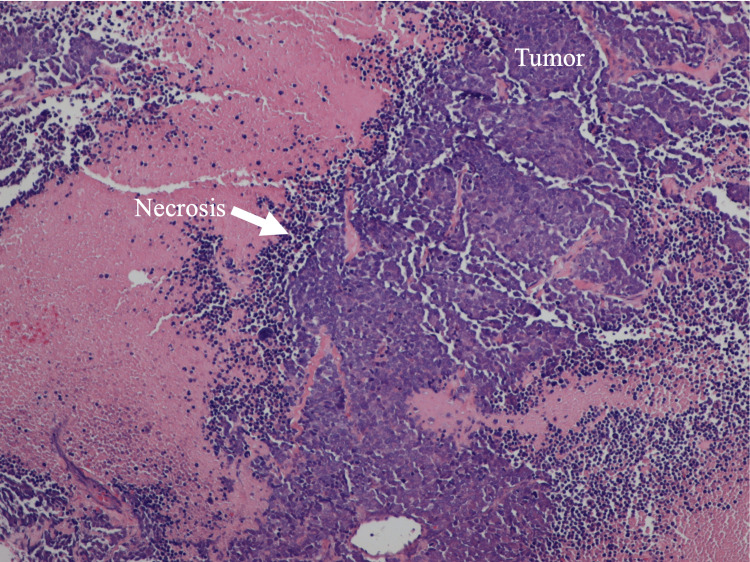
Histopathologic exam of biopsy specimen taken at low power (10x), highlighting tumor cells in clusters with necrosis.

**Figure 4:**
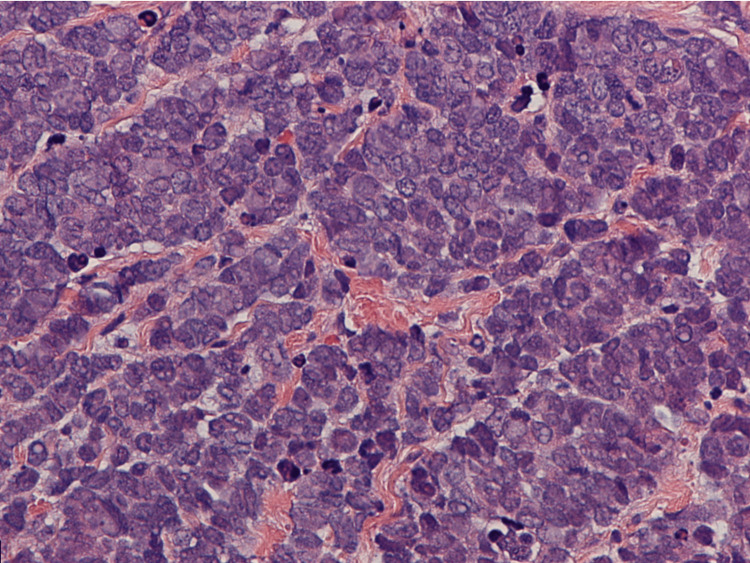
Histopathologic exam of biopsy specimen taken at high power (40x), showing closely packed nests of cells with round and uniform nuclei.

A 52-year-old female with a past medical history significant for diabetes, obesity, gastroesophageal reflux disease (GERD), obstructive sleep apnea (OSA), and depression presented to our clinic with concerns about a self-palpable mass in her left breast. A routine mammogram from five months prior to her noticing the mass was normal. A diagnostic mammogram and ultrasound of the left breast following presentation in August 2023 showed an area of focal asymmetry in the palpable abnormality (12:30 position, 4 cm from nipple) and a rounded hypoechoic lobulated focus with angulated margins measuring 4.1 x 2.5 x 3.4 cm on ultrasound (Figure 1). A biopsy of the mass in September 2023 showed neuroendocrine carcinoma, high grade with necrosis, otherwise classified as small cell carcinoma (Figures 2-4).

**Figure 5:**
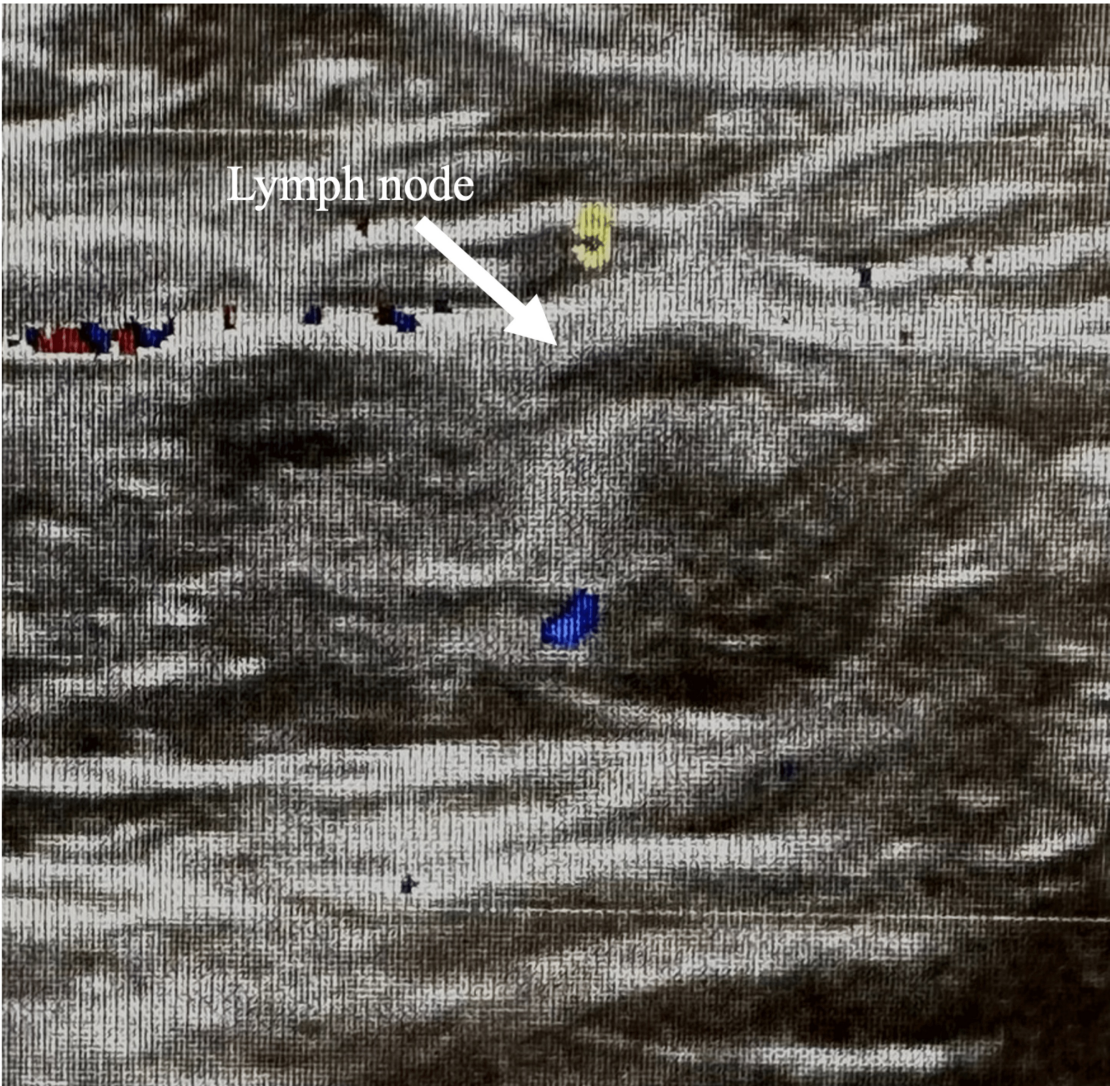
Ultrasound of left axilla shows thickened cortical margin to lymph node, suggesting malignancy.

Further analysis showed the mass to be estrogen receptor (ER) positive (31-40%), progesterone receptor (PR) negative, and human epidermal growth factor receptor 2 (HER2) negative, with a proliferative index (measured through Ki67 expression) of 95%. Ultrasound of the left axilla in September 2023 showed a thickened cortical margin to one of the lymph nodes, while subsequent biopsy of the lymph node revealed metastatic carcinoma (Figure 5).

**Figure 6:**
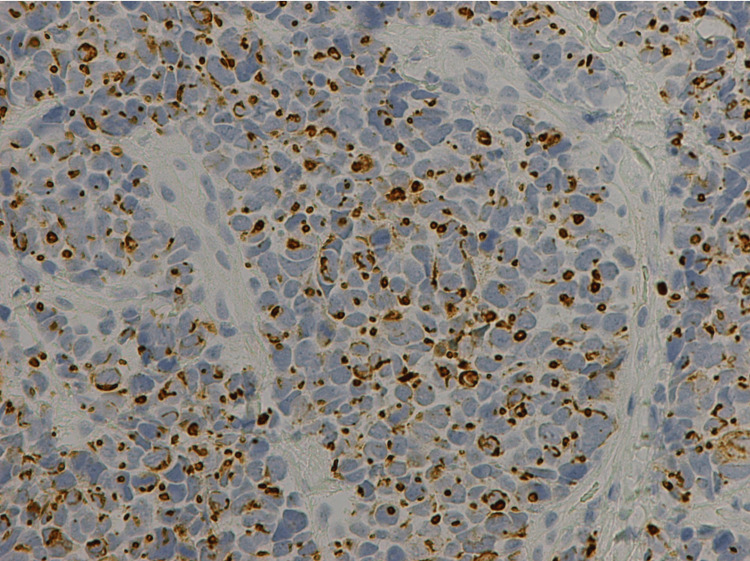
Immunostaining showing specimen positive for CK20, with a dot-like perinuclear staining pattern. CK20: cytokeratin 20.

**Figure 7:**
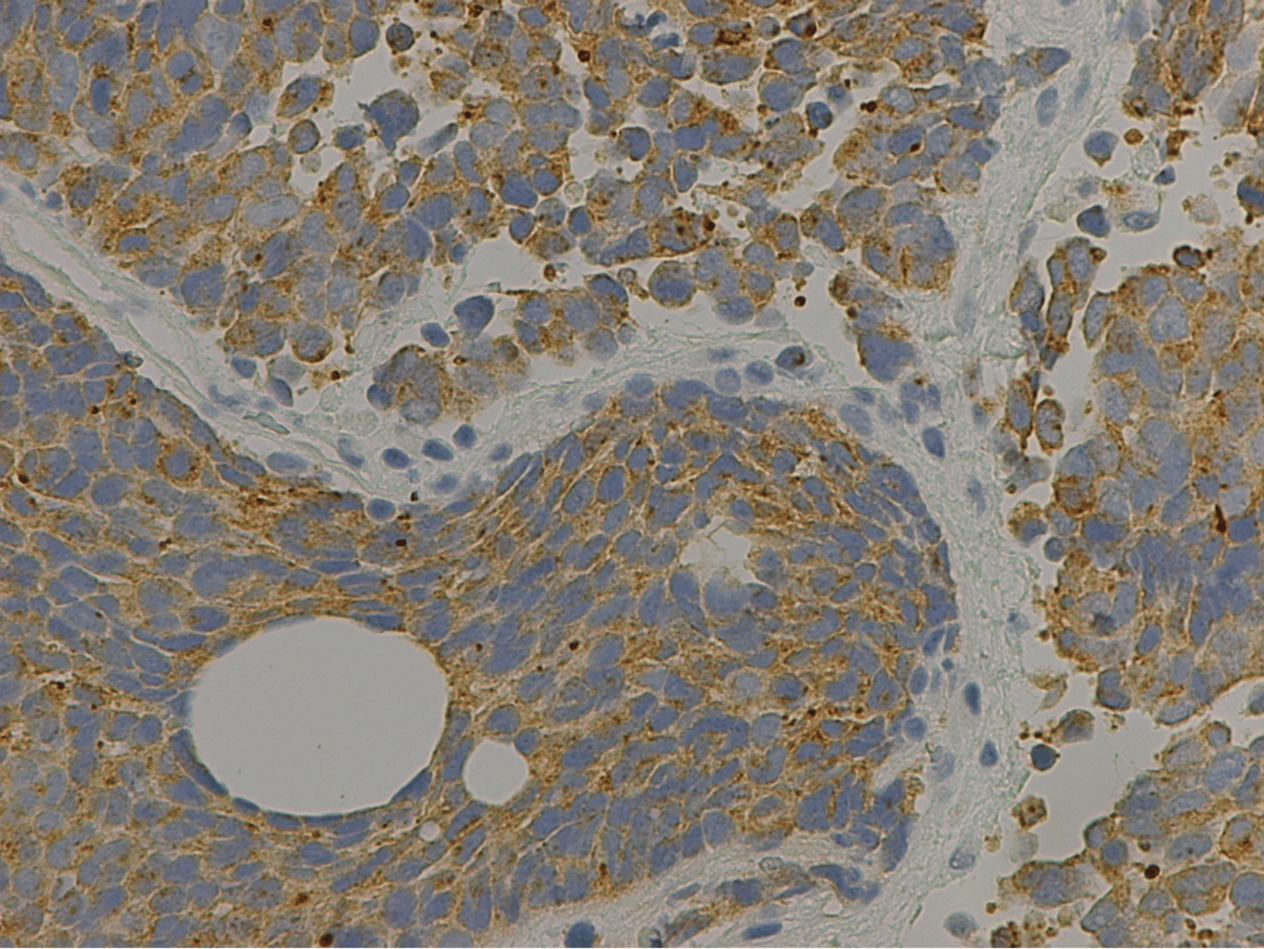
Immunostaining showing specimen positive for synaptophysin.

**Figure 8:**
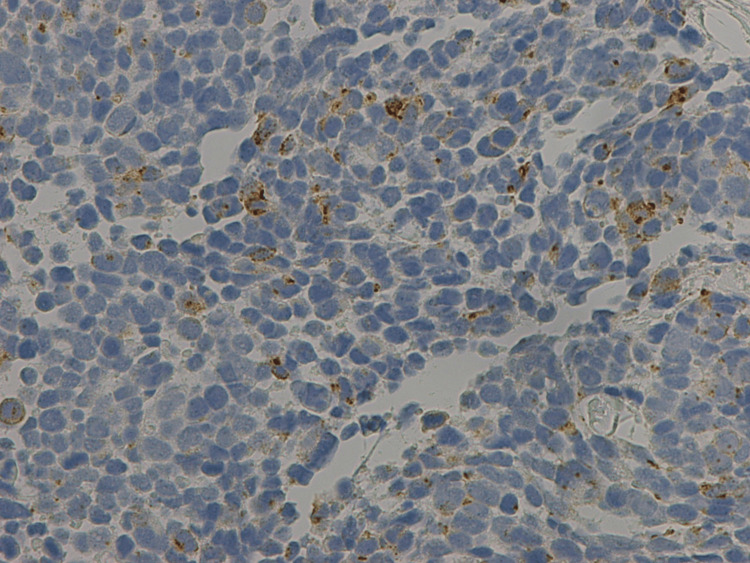
Immunostaining showing specimen positive for chromogranin A.

Immunostaining further validated the presence of small cell carcinoma, with stains showing the tumor to be positive for pan-keratin, cytokeratin 20 (CK20), synaptophysin, focally positive for chromogranin and thyroid transcription factor 1 (TTF-1), and negative for cytokeratin 7 (CK7) and GATA-3 (Figures 6-8). In particular, CK20 showed a distinctive dot-like perinuclear staining pattern, suggestive of Merkel cell carcinoma, although further immunohistochemistry analysis through the Mayo Clinic was negative for the Merkel polyomavirus oncoprotein. Additionally, the patient’s serology study was negative and there was no skin lesion or abnormality on exam that could have started as a Merkel cell.

Mayo Clinic then reviewed the tissue biopsy and confirmed neuroendocrine carcinoma. Prior to beginning chemotherapy for her neuroendocrine carcinoma, the patient underwent a pretreatment MRI of the brain, CT of the chest, abdomen, and pelvis, and a bone scan to rule out metastatic disease from elsewhere in her body (PET was initially refused through insurance). CT of the chest, abdomen, and pelvis showed an enhancing lobulated left breast mass measuring 5.5 x 4.2 cm and a prominent left axillary lymph node with a biopsy clip, which were both evident on previous imaging. Additionally, there was subtle heterogenous C6 vertebral body sclerosis, an indeterminate right adrenal gland lesion measuring 2.5 x 1.9 cm, and asymmetric enlargement of her left ovary. Bone scan was significant for increased uptake in the C6 region, corresponding with CT. For further evaluation, an MRI of the cervical spine was performed, which showed a diffuse C6 vertebral body and a new smaller round T2 and T3 vertebral body enhancing marrow edema concerning for metastatic disease. There were also heterogeneous non-enhancing marrow signals in the C5 vertebral body, right T1 lamina, and right T2 pedicle suggestive of hemangiomas. PET-CT was then ordered to further evaluate her abdomen following the CT results but was denied by insurance, necessitating an MRI. MRI of the abdomen further characterized the right adrenal gland lesion as a benign right adrenal adenoma. Biopsy of the C6 vertebral body was thought to be technically difficult, and later review of imaging through the Mayo Clinic suggested that the C6 finding likely had a benign hemangioma appearance.

Clinically, it was then determined that our patient had stage IIIA breast cancer (cT2, cN1, cM0, G3, ER+, PR-, HER2-). In mid-September 2023, she was started on systemic therapy consisting of carboplatin with etoposide and atezolizumab and had received three cycles by November 2023. While being evaluated on treatment, she did not have any significant clinical response in the breast. Given her treatment resistance, she required repeat imaging.

In November 2023, she had a consultation at Mayo Clinic and had a CT of the chest, abdomen, and pelvis, as well as a PET. PET/CT showed an intense fluorodeoxyglucose (FDG)-avid left breast mass at 12 o-clock, consistent with the known primary neoplasm, as well as an FDG-avid left axillary lymph node containing a biopsy clip. Additionally, there were indeterminate tiny FDG-avid foci in the liver, described as possibly artifactual, although tiny hepatic metastases could not be excluded. It was then decided to proceed with surgery at Mayo Clinic.

In December 2023, our patient underwent a left lumpectomy with left axillary lymph node dissection. Surgical pathology showed high-grade neuroendocrine carcinoma with Merkel cell features, forming a 9 cm mass and staged as pT3N1a. The 9 cm mass was removed with the closest margin < 1 mm in one area. Three out of 20 nodes were positive, with the largest measuring 4.1 cm, with extranodal extension. There was no definite response to presurgical treatment noted in the invasive cancer in the breast or the lymph nodes. Additionally, there was no identified dermal lymphatic or vascular invasion, and the cancer was identified as ER negative, PR positive (61-70%), and HER2 negative immunohistochemistry (IHC) 0. She was planned to receive capecitabine (Xeloda) and radiation after surgery; however, we witnessed a significant rapid downhill course post surgery.

**Figure 9:**
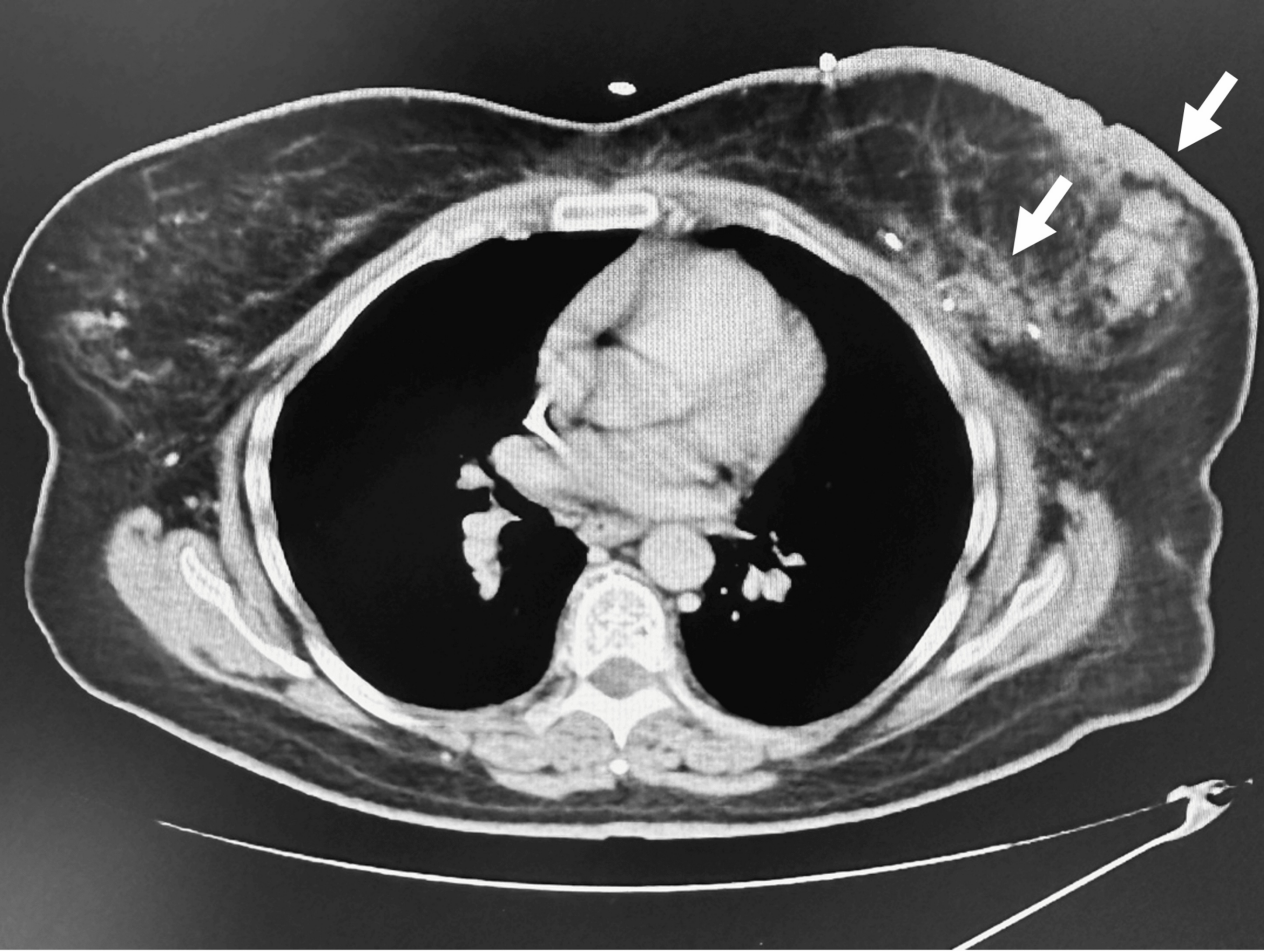
CT of the chest shows left breast thickening with multiple soft tissue metastases.

**Figure 10:**
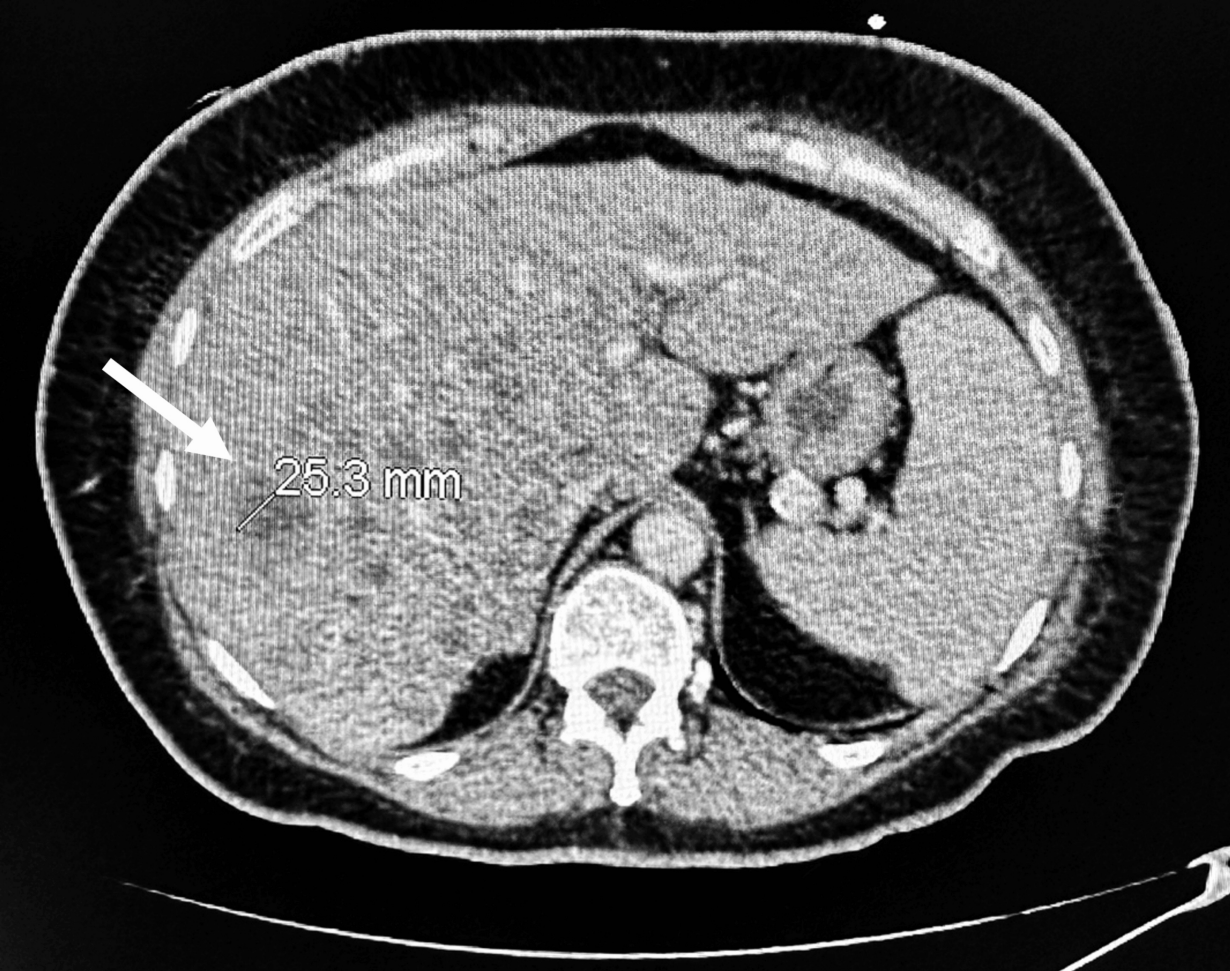
CT of the abdomen shows scattered low attenuating lesions throughout the liver, with the largest measuring close to 3 cm in size.

In January 2024, she underwent an emergent CT of the chest per pulmonary embolism (PE) protocol for evaluation of shortness of breath and tachycardia, which was negative for pulmonary embolism and pleural effusion. A week later, she was noted to have abnormal liver function tests, so a repeat CT of the chest, abdomen, and pelvis was completed. Notably, it showed marked thickening of the left breast with multiple soft tissue metastatic nodules (Figure 9). There was marked hepatic metastatic disease with innumerable scattered low attenuating lesions throughout the entire liver varying in size, with the largest in the right hepatic lobe measuring up to 3 cm (Figure 10).

**Figure 11:**
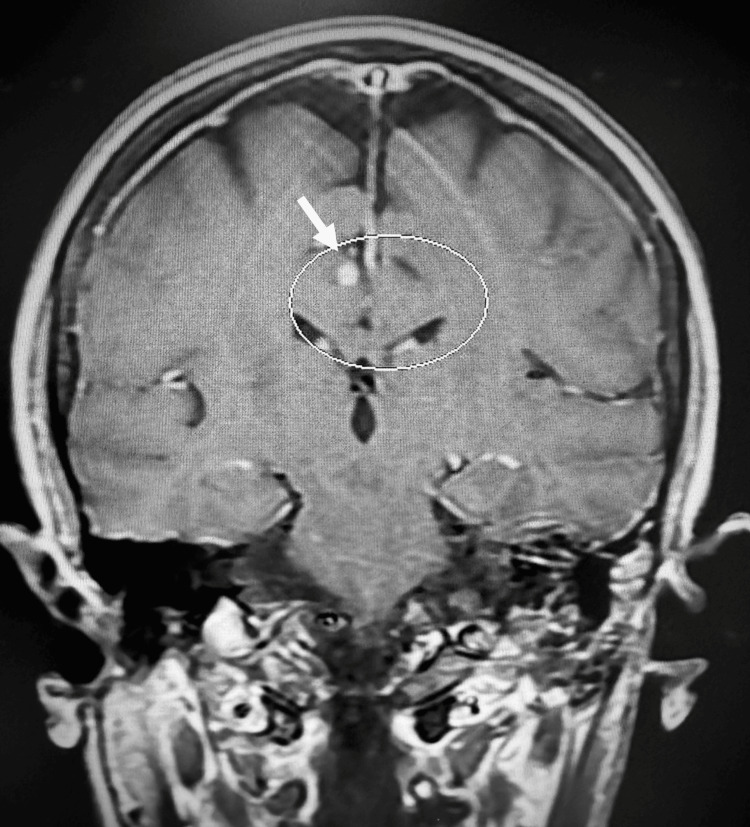
MRI of the head shows a new 3-mm enhancing lesion in the right frontoparietal parasagittal cortex.

**Figure 12:**
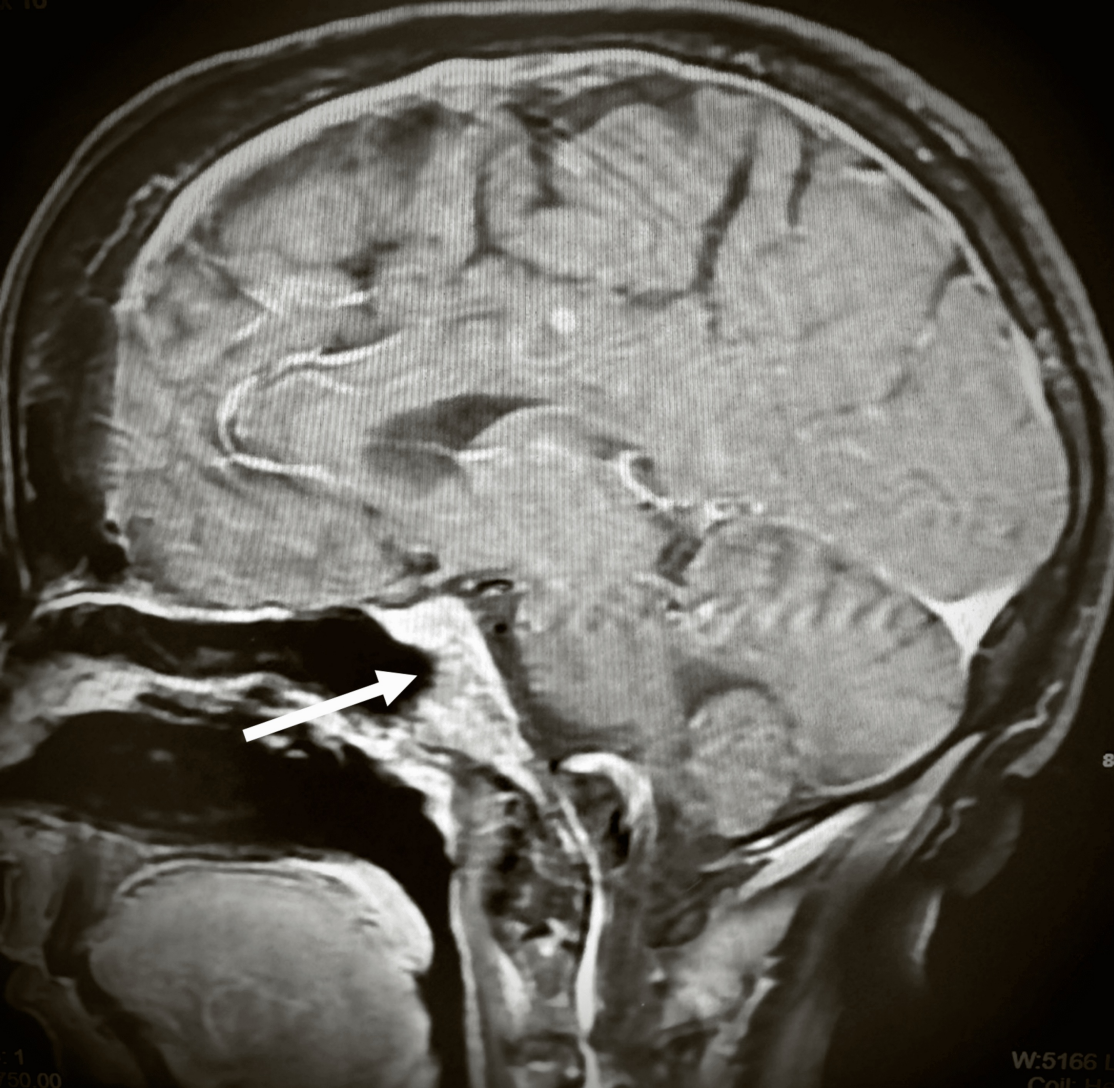
MRI of the head shows a 7 x 4 mm peripherally enhancing lesion within the clivus.

By mid-January 2024, the patient’s husband reported that she was confused and had begun to have cognitive issues. An urgent MRI of the head was completed, which showed a new 3 mm enhancing lesion in the right frontoparietal parasagittal cortex, likely representing a metastatic focus (Figure 11). A 7 x 4 mm peripherally enhancing lesion within the clivus suggestive of additional bony metastatic disease was also noted (Figure 12).

Within days, she had a rapid decline with worsening liver failure, hypercalcemia, and increasing confusion. She became unable to function independently and was hospitalized and seen by palliative care. It was then decided to focus on comfort measures with a transition to home hospice. Several days later, she passed away at home with family by her side.

Of note, the patient did have Tempus testing completed to assess for molecular markers that would have further guided treatment strategies; however, the results are no longer accessible.

## Discussion

Terminology and recommendations surrounding neuroendocrine carcinoma of the breast have changed over time. The first description of neuroendocrine differentiation in breast carcinoma was in 1963, with mucinous carcinomas of the breast showing positive silver staining [4]. In 1977, the first case series specific to NETs was published by Cubilla and Woodruff, along with the coining of the term "primary carcinoid of the breast" [5]. The first diagnostic guidelines for NETs of the breast were published by Sapino et al. in 2001, which described tumors with over a 50% expression of neuroendocrine markers (synaptophysin and chromogranin) as primary neuroendocrine tumors of the breast [6]. By 2003, the WHO recognized "neuroendocrine carcinoma of the breast" in their book *Pathology and Genetics of Tumours of the Breast and Female Genital Organs*, and Sapino’s prior diagnostic guidelines were accepted [7]. In the 2012 WHO Classification of Tumors of the Breast, "neuroendocrine carcinoma of the breast" was revised to "carcinomas with neuroendocrine features" and the prior 50% neuroendocrine marker positivity guideline was removed [8]. With the wide variability in classification and stepwise changes in defining NETs of the breast, it has become difficult to accurately determine the true incidence, with estimations ranging between 0.1% and 19.5% [9,10].

Clinically, the presentation of primary breast NETs is similar to other types of breast cancer, which makes diagnostic workup challenging [11]. Most often, patients present with a painless palpable retroareolar mass [12]. Patients may also notice nipple retraction or bloody nipple discharge [13]. Importantly, this kind of tumor affects older women as compared to traditional breast cancers, often occurring in their sixth or seventh decade of life [2].

On initial diagnostic imaging, breast NETs generally appear as heterogeneously dense oval or lobulated masses with unclear margins and will more often present as a mass instead of calcified lesions when compared to IDC-NOS [14]. Additionally, there is a similarity in mammography of NETs and triple-negative breast cancer [14].

Axillary metastases have been reported in between 43% and 47% of cases of primary breast NETs [15]. While there is no currently accepted method for grading, staging, and treating primary NETs of the breast, there have been recommendations for staging and treating such cancer like traditional breast cancers, with both stage and tumor location factoring into surgical treatment [15]. Alternatively, tumor grading has not shown clinical significance in recent WHO classifications, making workup and proper management even more difficult [8].

Current guidelines for managing neuroendocrine/small cell carcinoma from the National Comprehensive Cancer Network (NCCN) include an evaluation with multiphasic chest/abdomen/pelvis CT, or chest CT and abdomen/pelvis MRI, to exclude metastatic disease as the cause of the NET [16]. If indicated, brain MRI or CT with contrast may also be completed, along with additional imaging such as an FDG-PET CT. While there were a few areas of potential concern in our patient’s initial CT and MRI, a review of imaging was suggestive of benign lesions in both the right adrenal gland and C6 vertebral body, ruling out metastatic disease as the primary cause.

According to the NCCN, if the lesion is determined to be resectable, as was in our patient, there are several possibilities for treatment: resection plus adjuvant chemotherapy with possible radiation therapy, neoadjuvant chemotherapy with possible radiation followed by resection, chemotherapy alone, or definitive chemoradiation with cisplatin and etoposide or carboplatin and etoposide [16]. Our patient’s treatment followed these guidelines, as she received three cycles of neoadjuvant chemotherapy consisting of carboplatin with etoposide and atezolizumab, and although she showed no clinical improvement, she subsequently underwent resection of the known lesion in her breast and the involved lymph node. Although she was going to receive capecitabine and radiation following surgery, her condition declined. If our patient had improved following resection, clinical surveillance along with chest, abdomen, and pelvis imaging every 12 weeks for one year, and every six months thereafter would have been recommended.

Metastases are an important consideration when discussing primary NETs of the breast. The brain has been described as one of the most common areas for breast cancer to metastasize, with some studies reporting a 5.1% incidence rate in patients with breast cancer [17]. Additionally, NETs are known to commonly metastasize to the liver, which was seen in our patient through a CT showing scattered low attenuating lesions [18]. Given that her primary tumor quickly metastasized throughout her breast and to her liver and brain, NCCN treatment guidelines then expanded from chemotherapy alone, to include immunotherapy or targeted therapy. Our patient had undergone Tempus testing for subsequent targeted therapy, but her clinical decline ultimately did not allow for her to benefit from such treatment.

This report highlights the importance of continued documentation surrounding cases of primary NETs of the breast. More specifically, the clinical management and outcomes of such cases. It is unfortunate that while receiving the current standard of care based on NCCN guidelines, our patient continued to decline from her cancer and thus could not complete her chemotherapy and radiation. It is important that we take this particular case as an example of how quickly primary NETs of the breast can spread and use it to further guide clinical management in patients with similar presentations.

## Conclusions

In conclusion, primary NETs of the breast are a rare subtype of breast carcinoma often affecting postmenopausal women in their sixth or seventh decade of life. Upon diagnostic imaging, primary NETs of the breast show dense oval or lobulated masses with unclear margins, which is similar in appearance to triple-negative subtypes. It is common for patients to also present with axillary metastases, although there is currently no clearly established method for staging or grading NETs of the breast. Further imaging with CT or MRI of the chest, abdomen, and pelvis along with a bone scan is useful in excluding metastases to the breast as the primary cause. While primary NETs of the breast may present similarly to other breast cancers, they are a particularly aggressive subtype and do not have one specific treatment regimen, but rather several possibilities for treatment based on the NCCN guidelines.

There are several areas of research specific to NETs of the breast that need to be further studied to more effectively guide the management of patients with these tumors. Although there are currently several proposed treatment plans for NETs of the breast, there is no way to determine if the order of resection followed by chemotherapy, or neoadjuvant chemotherapy followed by resection is any more effective than chemotherapy alone or definitive chemoradiation, as there are so few documented cases in the literature to draw such conclusions. Most importantly, cases such as this one need to continue being published to exhibit the diagnosis, workup, and management that was completed, to inform future cases of similar patients, and hopefully avoid the same outcome.
